# A Novel Music Emotion Recognition Model Using Neural Network Technology

**DOI:** 10.3389/fpsyg.2021.760060

**Published:** 2021-09-28

**Authors:** Jing Yang

**Affiliations:** Zhejiang Conservatory of Music, Hangzhou, China

**Keywords:** emotion recognition, music, BP neural network, ABC algorithm, MediaEval Emotion in Music data set

## Abstract

Music plays an extremely important role in people’s production and life. The amount of music is growing rapidly. At the same time, the demand for music organization, classification, and retrieval is also increasing. Paying more attention to the emotional expression of creators and the psychological characteristics of music are also indispensable personalized needs of users. The existing music emotion recognition (MER) methods have the following two challenges. First, the emotional color conveyed by the first music is constantly changing with the playback of the music, and it is difficult to accurately express the ups and downs of music emotion based on the analysis of the entire music. Second, it is difficult to analyze music emotions based on the pitch, length, and intensity of the notes, which can hardly reflect the soul and connotation of music. In this paper, an improved back propagation (BP) algorithm neural network is used to analyze music data. Because the traditional BP network tends to fall into local solutions, the selection of initial weights and thresholds directly affects the training effect. This paper introduces artificial bee colony (ABC) algorithm to improve the structure of BP neural network. The output value of the ABC algorithm is used as the weight and threshold of the BP neural network. The ABC algorithm is responsible for adjusting the weights and thresholds, and feeds back the optimal weights and thresholds to the BP neural network system. BP neural network with ABC algorithm can improve the global search ability of the BP network, while reducing the probability of the BP network falling into the local optimal solution, and the convergence speed is faster. Through experiments on public music data sets, the experimental results show that compared with other comparative models, the MER method used in this paper has better recognition effect and faster recognition speed.

## Introduction

Music appeared earlier than language, and human beings are born with music to express feelings. Music plays an important role in the history of mankind, and music is integrated into all aspects of human life. With the development of science and technology, the creation, storage, and dissemination of music have all undergone tremendous changes. The changes in creation, storage, dissemination, and technology have made music play an increasingly important role in human social life. Stores often play dynamic music to arouse customers’ desire to buy. Playing rhythmic music during exercise can increase the enthusiasm of exercise and reduce exercise fatigue. Playing soft music during a break can soothe the nerves. Exciting and rhythmic music is played during the party, which can set off the joyful atmosphere of the party. During the medical process, appropriate music will be selected to provide psychological counseling to the patient. Music has been integrated into all aspects of life including waking up, eating, shopping, learning, sports, driving, education, medical care and so on. With the rapid increase of music file data, how to use computers to complete fast and effective music information retrieval has become a basic demand of people in the current society. Traditional music retrieval methods are based on classification tags, such as retrieving songs by song name, artist name, and album name. This method is currently the most commonly used method. The traditional methods of retrieving music information can no longer meet people’s needs for intelligent and personalized music retrieval (Casey et al., 2008). For these needs, many music websites have also launched music recommendation services. Recommend similar songs in the music library according to the needs of users and the songs that users often listen to. In order to obtain a better user experience, in recent years, various listening platforms have begun to provide song recommendation services with different moods. There are relatively few intelligent classification of music emotions and emotion-based search services. Therefore, emotion-based music retrieval is an important part of meeting people’s personalized music retrieval needs, and it is also an important development direction of current music retrieval.

To achieve emotion-based music classification and retrieval, it is necessary to label music works with emotions. Emotional annotation of massive music works based on artificial methods is not only a huge workload, but also the quality cannot be guaranteed. Therefore, it is inevitable to study music emotion automatic recognition technology and realize automatic emotion labeling of music works. Music emotion recognition (MER) refers to the construction of a calculation model based on music audio data and other related information to realize automatic music discrimination (Vidas et al., 2020). Agarwal and Om (2021) constructed a MER system to estimate the evaluation value of each musical work, and used regression methods to detect emotional changes in music. Lu et al. (2006) proposed a hierarchical MER system. The system uses Gaussian mixture model (GMM) and Bayesian classifier to classify music emotions. The identification object is popular music. This research is the first time that the GMM has been applied to music sentiment classification. Torres et al. (2007) identifies music emotions through music lyrics information. Sharaj et al. (2019) identified music emotions by analyzing auxiliary information such as lyrics in the music. The research also compares the recognition effects of three widely used recognition models: support vector machine (SVM), K-Nearest Neighbor Method (KNN), and GMM. Liu et al. (2006) uses the Psy Sound2 system they developed to extract music features from music waveform files. The classification algorithm is used to simulate the feature classifier and evaluate each feature to improve the reliability and robustness of the system, thereby obtaining music emotion. Wu and Jeng (2008) used the probability distribution model to measure the similarity between the tested music and the sample music based on the Hevner emotion model, so as to estimate the emotion type of the tested music. Anders (2011) expressed various common emotions by controlling the combination of multiple feature quantities such as the rhythm and tone of music. The relationship between feature quantity and music emotion was given by multiple music experts through manual annotation. Shan et al. (2009) applied MER to film music, and its goal was to judge the type of film based on the type of music emotion. Yang and Chen (2011) studied a music ranking algorithm based on a two-dimensional emotional model. Lin et al. (2011) uses online genre-labeled vocabulary for sentiment classification. Sentiment classification is performed after genre classification. Zhang and Sun (2012) conducts emotion recognition on web music and classifies music into a certain type of emotional label. The above studies are based on machine learning algorithms (Jiang et al., 2019, 2021), and the recognition effect can reach more than 60%.

In this study, back propagation (BP) neural network (Rumelhart et al., 1986) is selected as the basic recognition model. Aiming at the problems of BP network which are easy to fall into local minima, slow convergence speed and poor generalization ability, this paper uses the final value obtained by artificial bee colony (ABC) algorithm iteration as the weight and threshold of BP network. The optimized BP network greatly improves its global search ability, while reducing the probability of falling into a local optimal solution, and the convergence speed is faster. The optimized BP network has better music recognition capabilities. The main contributions of this article are:

(1) After comparative analysis, the BP neural network in machine learning is selected as the basic model of MER. After the BP neural network selects a large amount of random data to pass the characteristic function, it can backpropagate the output result with larger error. The characteristic function is sent through continuous feedback, and the cyclic operation can be performed again. After a large number of cyclic operations, the ideal value is output. This is the adaptive learning process of BP neural network. The BP neural network has become the most widely used artificial neural network model due to its strong non-linear mapping ability, high fault tolerance, self-adaptation and self-learning performance.
(2) Aiming at the problems of BP network that are easy to fall into local minima, slow convergence speed, and poor generalization ability, this paper uses the output obtained by the ABC algorithm as the weight and threshold of the BP network. This optimization method greatly improves the global search capability of the BP network, while reducing the probability of falling into a local optimal solution, and the network converges faster.
(3) To verify the effectiveness of the network used, based on the public music data set, four methods of SVM, KNN, GMM, and BP are used for experimental comparison. The experimental results show that the used network is better than other comparison models in terms of recognition rate and generalization.

## Theoretical Knowledge of Music Emotion Recognition

### Music Emotion Recognition Process

**FIGURE 1:**
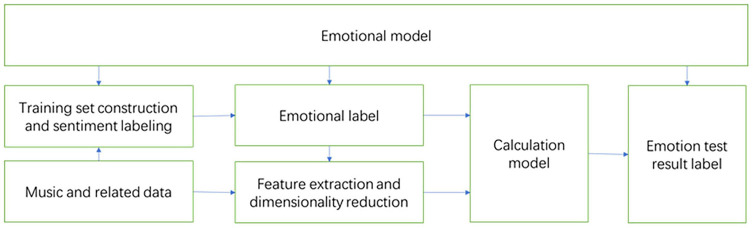
Music emotion recognition process.

The process of MER is shown in Figure 1. Almost all existing MER algorithms are based on supervised learning, so it is necessary to first establish a learning library, that is, a music data set with emotion annotations. Then extract the features of the music to form a feature vector, use the feature vector and emotion annotations for training, and get the recognition model. Then perform feature extraction on the unknown music, and input the extracted feature vector into the trained recognition model for recognition, so as to obtain the emotion classification result of the unknown music.

### Musical Emotion Model

**FIGURE 2:**
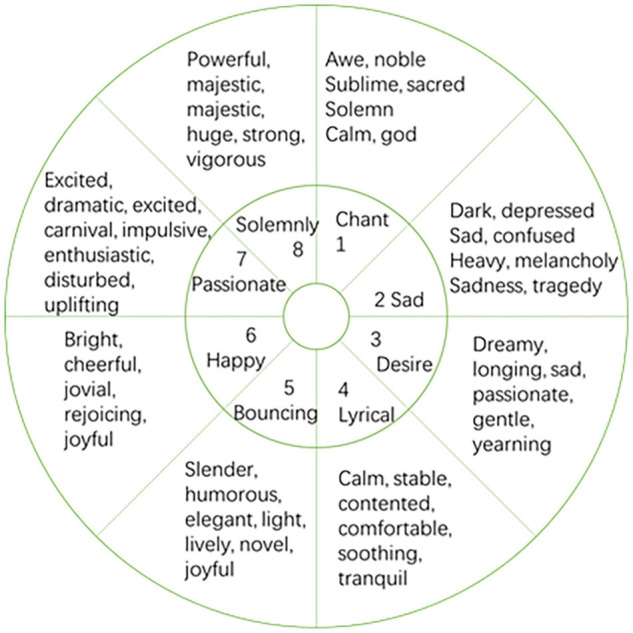
Hevner emotional model.

The premise of automatic recognition of music emotion is to establish a reasonable emotional psychological model. Due to the subjectivity of emotions, the modeling methods of musical emotion mental models are also different. Up to now, psychologists have proposed several classic emotional psychological models by studying the relationship between music and emotion. Commonly used are discrete emotion models and continuous emotion models. The discrete emotion modeling method divides several typical different emotion categories, and each category can contain a group of similar emotion descriptions. The continuous dimension model represents human emotional state as points in a two-dimensional or three-dimensional continuous space, which is a continuous emotional psychological description method. In the discrete emotion model, different adjectives are used to describe some basic emotions. Among them, the most famous is the Hevner emotional ring model (Hevner, 1936, 1937), which is shown in Figure 2. The Hevner model consists of 8 groups of 67 emotional adjectives arranged in circles. Among them, the adjectives in each large group express similar emotions, the emotions expressed by the adjacent large groups are closer and gradually progress, while the emotions expressed by the large groups in the relative position are just the opposite.

Compared with the discrete emotion model, the dimensional emotion space represents emotion through a small number of emotion dimensions. Dimensions are intended to correspond to the emotional representations within human beings. Continuous dimensional models usually represent human emotions as points in a multi-dimensional continuous space. Generally speaking, the higher the dimensionality, the more detailed the expression of emotions. But it is not that the higher the dimension, the better the emotion. If the dimensionality is too high, it will become very complicated, so the general dimensional model is two-dimensional or three-dimensional. The dimensional model does not simply classify human emotions, but describes the subtle differences between emotional states. It is a fine-grained emotional model. The disadvantage of the dimensional model is that there is a gap with people’s common cognitive methods, the interaction is not good, and it is very difficult to describe the emotions in detail. The widely used continuous dimensional model is the VA model.

**FIGURE 3:**
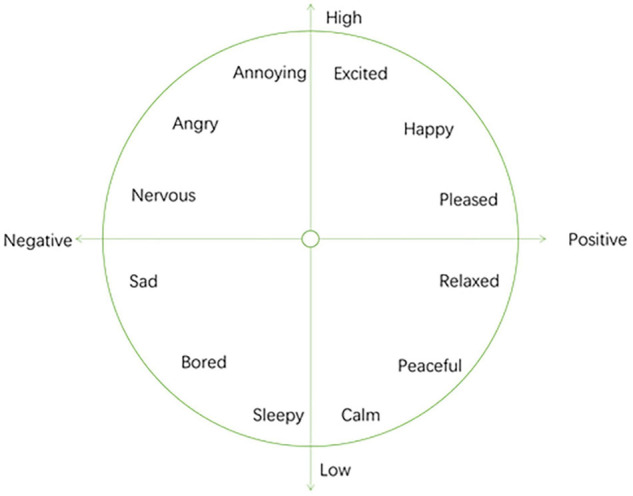
VA emotional model.

The VA model is proposed by Russell (Kanimozhi and Raj, 2015), as shown in Figure 3. This model abstracts emotions into a two-dimensional space based on arousal and valence. Valence’s term in psychology is called “inducing force,” which is a measure of whether people feel happy or not. The term arousal in psychology is called “activity,” which is a measure of whether people’s emotions are high. Russell found that the two variables valence and arousal can represent all emotional changes. She pointed out that people’s emotional preference is directly related to valence, but valence is not the only determining factor. Activation and people’s emotional preferences show an “inverted U-shape.” Her discovery can be summarized as the following three laws: (1) Valence has a linear relationship with people’s emotional preference. (2) Activation and people’s emotional preference are in an inverted U shape. (3) Valence interacts with Activation. When people are happy, there will be a higher activation value, and when people are unhappy, there will be a lower activation value. The positive and negative values of arousal and valence can be used to express four types of emotions: Positive valence and positive arousal means happiness and excitement. Negative valence and positive arousal means anger and anxiety. Negative valence and negative arousal means sadness and disappointment. Positive valence and negative arousal means relaxation and quietness.

### Music Data Set

In MER, currently commonly used public data sets mainly include Computer Audio Lab (CAL) (Hu et al., 2008), Music Information Retrieval Evaluation eXchange (MIRER) (Downie, 2008), MediaEval Emotion in Music (MEM) (Aljanaki et al., 2017), and AMG1608 (Chen et al., 2017).

Computer Audio Lab contains 500 Western pop music from different musicians. The emotion model used in this data set is a discrete emotion model. However, the 18 emotions included in the model are not completely mutually exclusive, and each emotion concept is marked as an integer value between 1 and 5. For the CAL data set, if the value corresponding to each emotion concept is binarized, a discrete model of emotion can be established. If the value corresponding to the emotion is regarded as a continuous value, then a dimensional model of the emotion can be established. It can be seen that a certain conversion can be carried out between the discrete emotion model and the dimensional emotion model.

Both MIRER 2007 AMC and MIRER 2013 K-POP AMC are derived from the MIREX evaluation activity and use the same emotional representation model. The evaluation activity began to classify music emotions in 2007. The data obtained through this evaluation activity has greatly promoted the development of MER technology.

MediaEval Emotion in Music is a dynamic MER algorithm evaluation. The data set used in this evaluation comes from the research and development results of Mohammad Soleymani and others. The data set used for the evaluation contains 1744 pieces of music, all of which are 45 s segments. Each segment is marked with a segment-level static VA value and a set of dynamic VA values with an interval of 0.5 s. The music sentiment labeling of this data set is done using Amazon Mechanical Turk, and each song has at least 10 people labeling. The dynamic VA value is marked in a continuous time mode and can be under-sampled as required. The track, audio, and emotion annotations of this dataset are completely public.

AMG1608 adopts the VA dimension model, and annotates a pair of VA values for each music segment. The labeling task of this data set is also completed by Amazon Mechanical Turk. This data set contains 1608 pieces of contemporary Western music, and each piece of music selects its most emotionally representative 30 s segment as the original audio data.

## Music Emotion Recognition Based on Improved Back Propagation Network

### Improved Back Propagation Network

**FIGURE 4:**
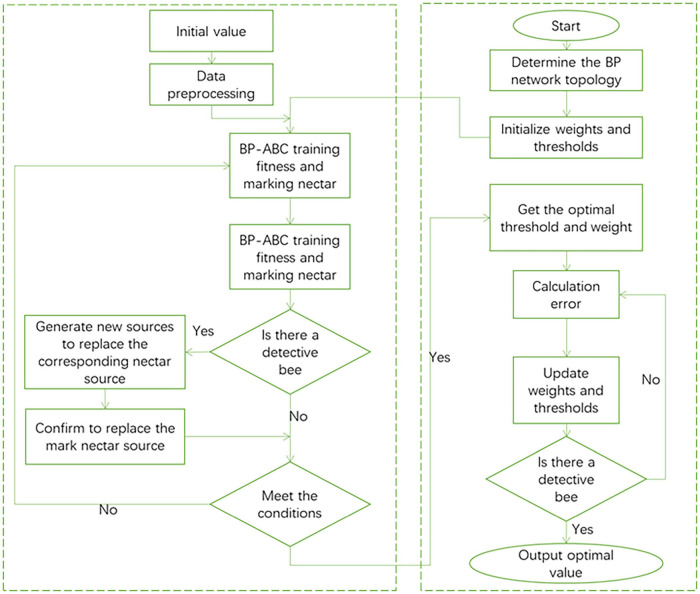
Improved BP network flow chart.

The introduction of the ABC algorithm (Zhou et al., 2021) improves the original BP neural network structure. The target output value of the ABC algorithm is used as the weight and threshold of the BP neural network. It can be seen that the ABC algorithm is mainly responsible for adjusting the weights and thresholds of the BP network, and feeds back the optimal weights and thresholds to the BP network. The BP neural network after the introduction of the ABC algorithm can greatly improve its global search ability, while reducing the probability of falling into a local optimal solution, and the convergence speed is faster. The execution process of the improved BP network is shown in Figure 4.

By introducing the ABC algorithm, the global search function of the BP neural network is greatly enhanced. In the BP network weight and threshold training, the ABC algorithm is used to match the optimal weight and threshold to the network connection of each neuron in the BP network. The original BP neural network uses gradient descent to train the weights and thresholds of the network. The method used in this article is to complete the adjustment of the weights and thresholds of the network with the cooperation of hire bees, follow bees and detective bees.

The ABC algorithm has a memory function. The original BP neural network is prone to redundant operations such as repeated operations, so the BP neural network is unstable. After each iteration of the improved BP algorithm, the algorithm will calculate the fitness of the solution. Calculate the fitness evaluation value, sort, and record the fitness value. After each iteration is completed, the output solution will be compared with the previous optimal solution. Take better solutions as records and save them to get the global optimal solution. In this way, problems such as repeated training caused by the unstable memory of the BP neural network are avoided.

The ABC algorithm adopts the survival of the fittest mechanism to solve the problem of handling superior solutions and inferior solutions. The ranking is performed according to the fitness value of the solution. The higher the fitness value of the solution, the higher the ranking level and the higher the probability of being selected. Finally, two iterations are selected to form the next generation group. Eliminate inferior solutions and combine high-quality solutions into the next-generation iteration group to speed up the algorithm’s convergence.

The algorithm implementation steps can be summarized as the following:

(1) Set the basic parameters of the BP neural network, such as the number of input nodes, the number of output nodes, the number of hidden layers, and the number of hidden layer nodes.
(2) Set the basic parameters of the ABC algorithm, such as the value of the nectar source, the size of the bee colony, and the maximum number of iterations.
(3) Randomly assign nectar sources to the hired bees, and the hired bees begin to collect nectar source information.
(4) Calculate the fitness of the nectar source and evaluate the fitness value of the nectar source.
(5 ) The operation follows the bee phase. Follow bees select nectar sources according to the rule of survival of the fittest and search for high-quality nectar sources in the neighborhood.
(6) The output optimal value is compared and judged with the original value, the optimal solution is retained and the number of finding solutions is updated. If the number of search times reaches the preset upper limit and the optimal value has not been updated, the search for nectar will not continue. The follower bee turns into a scout bee and starts searching for new sources of nectar.
(7) The reconnaissance bee operation stage. Determine whether the number of iterations is the maximum. When the maximum number of iterations is not reached, jump to (4) to continue execution.
(8) Pass the global optimal solution obtained by the ABC algorithm to the BP neural network as the optimal weight and threshold solution. BP neural network uses the obtained optimal weights and thresholds for data training.

### Music Emotion Recognition Process Based on Improved Back Propagation Network

**FIGURE 5:**
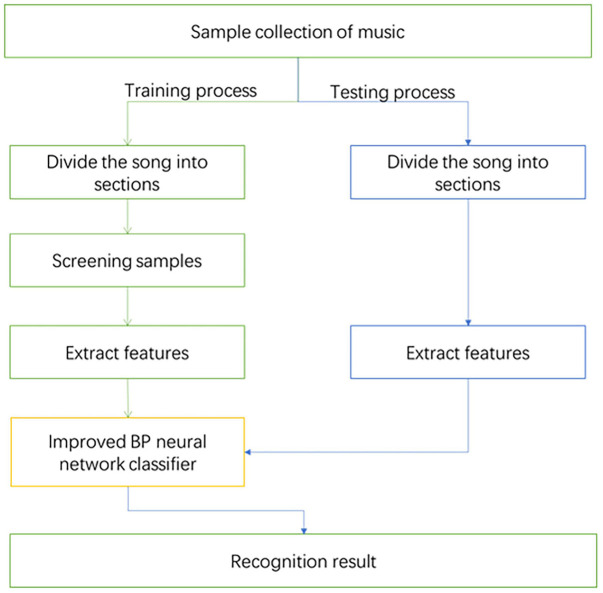
Identification process.

First, select the samples needed for the experiment in this article, and divide the samples into two parts: training samples and test samples. The training samples are used as initial data to train the MER model. Then use test samples to detect the correctness of the training process, so as to realize MER. The experimental process is shown in Figure 5.

## Analysis of Results

### Experimental Data

The experimental data set of this study is the MEM data set, 80% of the entire data set is selected as the training set, and the remaining 20% is used as the test set. The music feature data is extracted and combined to construct training and testing of MER. In order to find a better combination of eigenvalues for MER, this experiment tries to combine the eigenvalues of the time domain, frequency domain, and cepstrum domain into multiple combinations to perform MER through the improved BP algorithm. Table 1 shows Different combinations of features.

**TABLE 1:** Details of feature combinations.

**No.**	**Features included in the combination**
1	Short-term energy, short-term average amplitude, short-term autocorrelation function, frequency spectrum, amplitude spectrum, phase spectrum, and complex cepstrum
2	Short-term energy, short-term average amplitude, short-term autocorrelation function, short-term zero-crossing rate, frequency spectrum, amplitude spectrum, and phase spectrum
3	Short-term energy, short-term average amplitude, short-term autocorrelation function, frequency spectrum, amplitude spectrum, phase spectrum, complex cepstrum, and cepstrum
4	Short-term autocorrelation function, frequency spectrum, amplitude spectrum, phase spectrum, and cepstrum
5	Complex cepstrum and cepstrum
6	Short-term energy, short-term average amplitude, short-term autocorrelation function, short-term zero-crossing rate, complex cepstrum, and cepstrum

### Evaluation Index

The contrast model is SVM (Duan et al., 2012), K Nearest Neighbor Algorithm (KNN) (Bressan and Azevedo, 2018), GMM (Wang et al., 2015), BP Neural Network (Di and Wu, 2015). The evaluation indicators of each recognition model are mean absolute error (MAE), root mean square error (RMSE), and square coefficient (*R*^2^). MAE and RMSE are error functions. The smaller the value of the function, the better the performance of the model. *R*^2^ represents the fit of the model. The fit is the overall curve between the predicted value and the actual value of the model, which represents the fitness of the regression model. The larger the *R*^2^ value, the closer the predicted curve is to the actual data curve, and the better the model performance.


(1) $$M\,A\,E=\frac{\sum_{i=1}^{N}\left|x_{i}-y_{i}\right|}{N}$$

(2) $$R\,M\,S\,E=\sqrt{\frac{\sum_{i=1}^{N}{\left(x_{i}-y_{i}\right)}^{2}}{N}}$$

(3) $$R^{2}=1-\frac{\sum_{i=1}^{N}{\left({\hat{z}}_{i}-z_{i}\right)}^{2}}{\sum_{i=1}^{N}{\left(z_{i}-{\bar{z}}_{i}\right)}^{2}}$$

(4) $$A\,\text{cc}=\frac{\mathrm{TP}+\mathrm{TN}}{\mathrm{TP}+\mathrm{FN}+\mathrm{FP}+\mathrm{TN}}$$

where, *x*_*i*_ and *y*_*i*_ are the predicted label and actual label of sample *i*. ${\hat{z}}_{i}$ and *z*_*i*_ are the predicted label and the actual label of the test sample *i*, and $\bar{z}$ is the average of the actual label of the test set. TP indicates that the positive sample is judged to be a positive class. TN indicates that the negative sample is judged as negative. FP indicates that the negative samples are judged as positive. FN indicates that the positive sample is judged as a negative class.

The processor of the computer used in the experiment is Intel^®^ Core^TM^ i5-6200U CPU @ 2.40 GHz, the memory is 12G, and the operating system is win10 64-bit. The software environment used is MATLAB 2019.

### Analysis of Results

Realize the task of MER as a classification task. The continuous emotions in VA emotional space are divided into four discrete categories, which are happy, sad, nervous and calm. Since the labels of the music clips in the data set are the points marked in the VA space, to map the emotional value to the emotional category, the emotional value needs to be divided. In this paper, the VA space is divided into four parts, and the four emotions are, respectively, corresponded to the VA space, and then the sample data is processed by classification tasks. In order to analyze which feature combination obtains the best recognition effect, the BP algorithm is first used to compare various combination features. Table 2 gives the experimental results of recognition rate.

**TABLE 2:** Music recognition rate of various feature combinations based on BP algorithm.

**Combination number**	**1**	**2**	**3**	**4**	**5**	**6**
Acc(%)	77.89	83.83	76.48	74.20	69.42	73.91

It can be seen from Table 2 that in the MER experiment based on BP, the feature combination with better recognition effect is composed of short-term energy, short-term average amplitude, short-term autocorrelation function, short-term zero-crossing rate, frequency spectrum, and amplitude spectrum. The correct rate is 83.83% for the feature combination composed of phase spectrum. The accuracy of the feature combination composed of short-term energy, short-term average amplitude, short-term autocorrelation function, frequency spectrum, amplitude spectrum, phase spectrum and complex cepstrum is 77.89%. The accuracy of the feature combination composed of short-term energy, short-term average amplitude, short-term autocorrelation function, frequency spectrum, amplitude spectrum, phase spectrum, complex cepstrum, and cepstrum features is 76.48%. It can be seen from the experimental results that temporal features have a good influence on MER.

**FIGURE 6:**
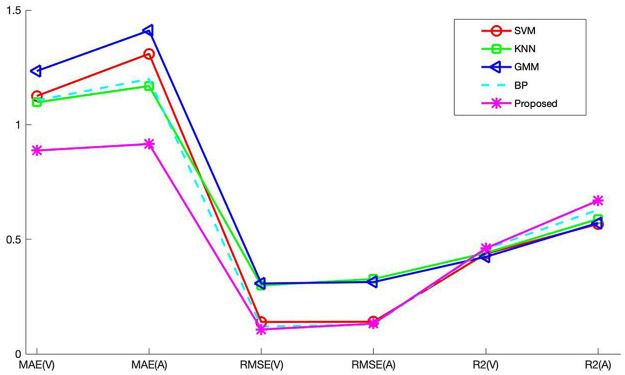
Comparison chart of recognition results.

Through the above experiment, it can be analyzed that the recognition result obtained by the feature data of Combination 2 is the best. Therefore, the music recognition rate of each model pair is compared based on the feature data of Combination 2. The recognition results of each recognition model on music data are shown in Table 3. Since the experimental results obtained by the recognition models are not much different, the changes are not obvious. In order to visually compare the experimental results, the experimental results are displayed in a line graph. Figure 6 is a comparison diagram of experimental results.

**TABLE 3:** Experimental results of each recognition model.

**Algorithm**		**MAE**	**RMSE**	***R* ^2^**
SVM	Valence	1.1253	0.1392	0.4374
	Arousal	1.3091	0.1405	0.5663
KNN	Valence	1.0974	0.2988	0.4403
	Arousal	1.1686	0.3265	0.5881
GMM	Valence	1.2342	0.3072	0.4236
	Arousal	1.4105	0.3136	0.5712
BP	Valence	1.1066	0.1190	0.4576
	Arousal	1.1987	0.1284	0.6284
Proposed	Valence	0.8872	0.1066	0.4606
	Arousal	0.9156	0.1322	0.6687

From the experimental results in Table 3 and Figure 6, the following conclusions can be drawn:

(1) The two index values of MAE and RMSE of each comparison model show that the value on the Valence dimension is lower than the value on the Arousal dimension. This shows that the results obtained in the Valence dimension are better. The value of the Valence dimension in the *R*^2^ indicator is lower than the value of the Arousal dimension. Since the larger the *R*^2^ parameter, the better the experimental result, so the Arousal dimension has a better effect on *R*^2^.
(2) The emotion recognition results obtained by the BP model are better than those of SVM, KNN, and GMM, which is why this article chooses BP as the basic recognition model. There are still some gaps between the recognition results of BP network and the proposed algorithm. This shows that the introduction of the ABC algorithm to optimize the BP network has a significant effect. The optimized BP algorithm can effectively improve the music recognition rate.
(3) The proposed method has little difference between the two dimensions of Valence and Arousal in MAE and RMSE index values. The difference between MAE and RMSE is 0.284 and 0.0256, respectively, indicating that the overall predicted value of the method used is very close to the actual value.

## Conclusion

The continuous development of modern information technology has also promoted audio digitization research. The use of computer-related technology for MER has gradually become a research hotspot. In order to improve the recognition rate of music emotion, this research uses an improved BP network to recognize music data. This research first classifies the acoustic features of music in a combined form for emotion classification, and analyzes the most suitable feature data for emotion recognition. The experimental results show that the classification of music features based on short-term energy, short-term average amplitude, short-term autocorrelation function, short-term zero-crossing rate, frequency spectrum, amplitude spectrum and phase spectrum is better. Secondly, a music sentiment classifier was constructed using the BP network optimized by the ABC algorithm and compared with the experimental results of other classifiers. Based on the experimental results, it can be seen that the network used has a better recognition effect. The improved BP network used can complete the emotion recognition of music. In the recognition process, it can also be found that the selection of music features is also an important factor that affects the recognition effect. Reasonable extraction of music features is also an important research content of emotion recognition. In the future, this research intends to apply deep learning methods to MER. Deep learning is a learning method based on feature hierarchical structure that can learn unsupervised features. It has the feature learning ability of artificial neural network. The learned features describe the data to a higher degree. Mass music is used for feature learning, so that the machine can independently select better music features to describe the relationship between music and emotion.

## Data Availability Statement

Publicly available datasets were analyzed in this study. This data can be found here: https://cvml.unige.ch/databases/emoMusic/.

## Author Contributions

JY completed the design of the experiment and the writing of the manuscript.

## Conflict of Interest

The author declares that the research was conducted in the absence of any commercial or financial relationships that could be construed as a potential conflict of interest.

## Publisher’s Note

All claims expressed in this article are solely those of the authors and do not necessarily represent those of their affiliated organizations, or those of the publisher, the editors and the reviewers. Any product that may be evaluated in this article, or claim that may be made by its manufacturer, is not guaranteed or endorsed by the publisher.
